# Deprescribing preventive cardiovascular medication in patients with predicted low cardiovascular disease risk in general practice – the ECSTATIC study: a cluster randomised non-inferiority trial

**DOI:** 10.1186/s12916-017-0988-0

**Published:** 2018-01-11

**Authors:** Clare H. Luymes, Rosalinde K. E. Poortvliet, Nan van Geloven, Margot W. M. de Waal, Yvonne M. Drewes, Jeanet W. Blom, Nynke Smidt, Willem J. J. Assendelft, Wilbert B. van den Hout, Wouter de Ruijter, Mattijs E. Numans

**Affiliations:** 1 Department of Public Health and Primary Care, Leiden University Medical Centre, PO Box 9600, 2300 RC Leiden, The Netherlands; 2 Department of Medical Statistics and Bioinformatics, Leiden University Medical Centre, PO Box 9600, 2300 RC Leiden, The Netherlands; 3 Department of Internal Medicine, Section of Gerontology and Geriatrics, Leiden University Medical Centre, PO Box 9600, 2300 RC Leiden, The Netherlands; 4 Department of Epidemiology, University Medical Centre Groningen, PO Box 30.001, 9700 RB Groningen, The Netherlands; 5 Department of Primary and Community Care, Radboud University Medical Centre, PO Box 9101, 6500 HB Nijmegen, The Netherlands; 6 Department of Medical Decision Making & Quality of Care, Leiden University Medical Centre, PO Box 9600, 2300 RC Leiden, The Netherlands; 7 Dutch College of General Practitioners, PO Box 3231, 3502 GE Utrecht, The Netherlands

**Keywords:** General practice, Preventive medicine, Cardiovascular disease, Antihypertensive agent, Anticholesteremic agent, Inappropriate prescribing

## Abstract

**Background:**

The use of cardiovascular medication for the primary prevention of cardiovascular disease (CVD) is potentially inappropriate when potential risks outweigh the potential benefits. It is unknown whether deprescribing preventive cardiovascular medication in patients without a strict indication for such medication is safe and cost-effective in general practice.

**Methods:**

In this pragmatic cluster randomised controlled non-inferiority trial, we recruited 46 general practices in the Netherlands. Patients aged 40–70 years who were using antihypertensive and/or lipid-lowering drugs without CVD and with low risk of future CVD were followed for 2 years. The intervention was an attempt to deprescribe preventive cardiovascular medication. The primary outcome was the difference in the increase in predicted (10-year) CVD risk in the per-protocol (PP) population with a non-inferiority margin of 2.5 percentage points. An economic evaluation was performed in the intention-to-treat (ITT) population. We used multilevel (generalised) linear regression with multiple imputation of missing data.

**Results:**

Of 1067 participants recruited between 7 November 2012 and 18 February 2014, 72% were female. Overall, their mean age was 55 years and their mean predicted CVD risk at baseline was 5%. Of 492 participants in the ITT intervention group, 319 (65%) quit the medication (PP intervention group); 135 (27%) of those participants were still not taking medication after 2 years. The predicted CVD risk increased by 2.0 percentage points in the PP intervention group compared to 1.9 percentage points in the usual care group. The difference of 0.1 (95% CI -0.3 to 0.6) fell within the non-inferiority margin. After 2 years, compared to the usual care group, for the PP intervention group, systolic blood pressure was 6 mmHg higher, diastolic blood pressure was 4 mmHg higher and total cholesterol and low-density lipoprotein-cholesterol levels were both 7 mg/dl higher (all *P* < 0.05). Cost and quality-adjusted life years did not differ between the groups.

**Conclusions:**

The results of the ECSTATIC study show that an attempt to deprescribe preventive cardiovascular medication in low-CVD-risk patients is safe in the short term when blood pressure and cholesterol levels are monitored after stopping. An attempt to deprescribe medication can be considered, taking patient preferences into consideration.

**Trial registration:**

This study was registered with Dutch trial register on 20 June 2012 (NTR3493).

**Electronic supplementary material:**

The online version of this article (doi:10.1186/s12916-017-0988-0) contains supplementary material, which is available to authorized users.

## Background

Cardiovascular disease (CVD) remains a leading cause of mortality and morbidity worldwide and is associated with a loss of quality of life and high costs [1, 2]. Physicians use their clinical judgement as well as clinical practice guidelines to determine whether treatment with antihypertensive and lipid-lowering drugs is necessary for individual patients. Recommendations concerning the initiation of drug treatment in patients with hypertension or hypercholesterolemia, but without established CVD, are subject to change and are still under debate. Currently, guideline recommendations concerning initiation of drug treatment are often based on composite risk scores [3–7]. However, recommendations to start medication in previous guidelines used to be based on single risk factors, such as increased blood pressure or cholesterol levels, or diabetes, and thus lacked an integrated approach to risk management [8–10], which resulted in drug prescription to patients who are now considered low-CVD-risk patients. Over time, these evolving recommendations have resulted in the potentially inappropriate use of antihypertensive and lipid-lowering drugs, namely, when potential risks (e.g., side effects) outweigh the potential benefits [11–14]. Although physicians are aware that medication use in low-CVD-risk patients is of little benefit, fear of negative consequences and lack of evidence for withdrawal keep them from stopping the medication [15]. A study investigating the positive (e.g., quality of life) and negative effects (e.g., increase in CVD risk and experiencing inconvenient symptoms) of deprescribing preventive cardiovascular medication in low-CVD-risk patients may improve physicians’ knowledge. Depending on the outcome, this may lead to a more positive or negative attitude towards deprescribing in this patient population amongst physicians. Therefore, the aim of the Evaluating Cessation of STatins and Antihypertensive Treatment In primary Care (ECSTATIC) study was to evaluate whether an attempt to deprescribe preventive cardiovascular medication in low-CVD-risk patients using these medications without indications according to current guidelines is safe and cost-effective.

## Methods

### Study design

The ECSTATIC study was designed and carried out as a cluster randomised non-blinded parallel-group active-control non-inferiority study, including patients from 46 general practices in the western part of the Netherlands from 7 November 2012, with a follow-up period lasting until 20 November 2015 (Dutch Trial Register, NTR3493). To reduce contamination of the participants in the control group, the unit of randomisation and analysis was the general practice. The primary outcome was the difference in the increase in the predicted 10-year CVD risk in the 2 years after the first visit. Our choice for a non-inferiority trial design was based on the expectation that the attempt to deprescribe preventive cardiovascular medication in low-CVD-risk patients would increase CVD risk to some extent, but, at the same time, would lead to fewer side effects, lower costs and the disutility of daily medication use, together tipping the risk-benefit ratio into its favour.

The study protocol was approved by the medical ethics committee of Leiden University Medical Centre. The study was conducted in accordance with the Declaration of Helsinki. The study received external funding from ZonMw, the Netherlands Organisation for Health Research and Development (reference 200320017). The funder of the study had no role in study design, data collection, data analysis or interpretation to the data.

To avoid allocation bias and imbalance in the number of general practices allocated to the study groups, we used computer-generated block randomisation in a 1:1 ratio, with random block sizes consisting of 10 or 12 general practices.

### General practices and participants

**Fig. 1:**
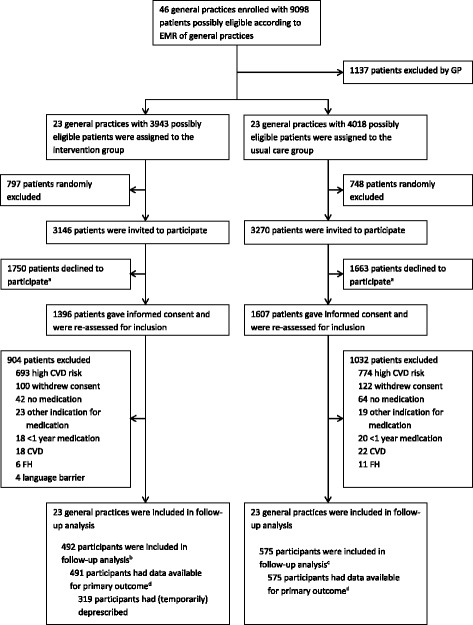
Trial profile. ^a^The number of patients who declined to participate did not differ between the intervention and usual care groups after adjusting for cluster randomisation (*P* = 0.28). ^b^At the measurement 3 months after the first visit, 459 participants had complete data available for calculation of the 10-year CVD risk score. At the measurement 24 months after the first visit, 403 participants had complete data available for calculation of the 10-year CVD risk score. ^c^At the measurement 3 months after the first visit, 546 participants had complete data available for calculation of the 10-year CVD risk score. At the measurement 24 months after the first visit, 499 participants had complete data available for calculation of the 10-year CVD risk score. ^d^Missing values of (systolic blood pressure and/or total cholesterol/HDL-cholesterol ratio and/or smoking status) of 88 participants in the intervention group and 76 in the usual care were imputed. One participant in the intervention group died of an unknown cause without having attempted to have her medication deprescribed and was not included in the analysis. CVD cardiovascular disease, EMR electronic medical records, FH familial hypercholesterolemia, GP general practitioner, HDL high-density lipoprotein

All general practices in our network were invited. Before randomisation, general practitioners (GPs) of the practices selected possibly eligible patients who were 40 to 70 years old without established CVD and who had been using potentially inappropriate antihypertensive or lipid-lowering drugs for at least 1 year based on their electronic medical record (EMR) (Fig. 1). Patients aged below 40 years old or over 70 years old were excluded because the SCORE risk function (recalibrated for the Dutch population), which we used to assess eligibility for inclusion, is available only for patients aged 40 to 70 years old [4]. Subsequently, the participating general practices were randomised. GPs sent a written invitation for trial participation to their patients who had already been declared eligible before randomisation. We used a complete-double consent design in which informed consent was sought in both the intervention group and usual care group, mentioning the use of the other comparison group. To avoid contamination of the usual care group, the invitation letter sent to the usual care group did not specify the exact intervention. The letter sent to the intervention group explained the intervention and mentioned the use of a control group that was given care as usual [16].

**Fig. 2:**
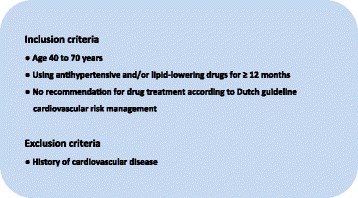
Inclusion and exclusion criteria for ECSTATIC trial

After obtaining informed consent from the patients, the researchers reassessed the patients for eligibility using the SCORE risk function recalibrated for the Dutch population as used in the Dutch guideline for Cardiovascular Risk Management (Fig. 2) [4]. An overview of all patient inclusion and exclusion criteria is listed in Additional file 1. We assessed the pre-treatment CVD risk based on current (i.e., at first visit) age, sex and smoking behaviour (smoking yes/no), in combination with reported pre-treatment systolic blood pressure (SBP) and total cholesterol/high-density lipoprotein (HDL)-cholesterol ratio levels in general practice EMRs. If these values were not available for up to 1 year before the start of drug treatment, pre-treatment SBP was conservatively estimated at 180 mmHg, and low-density lipoprotein (LDL) and high-density lipoprotein (HDL) cholesterol levels were estimated based on current levels of total cholesterol, HDL-cholesterol and LDL-cholesterol measured by local laboratories (Additional file 1).

The Dutch College of General Practitioners provided us with the underlying algorithm for CVD risk estimation. We used this algorithm to estimate predicted 10-year CVD risk. All patients willing to participate who had a predicted low 10-year risk of CVD morbidity and mortality, without additional risk increasing factors (further reported as low CVD risk), i.e., patients for whom drug treatment was not recommended according to the Dutch guideline for Cardiovascular Risk Management, were included in the trial [4].

### Interventions

GPs and (when applicable) practice nurses in intervention practices received a 2-hour workshop providing information about the background, the aim and the intervention of the ECSTATIC study. The workshop was carried out in Leiden University Medical Centre and led by a GP with a special interest in cardiovascular risk management and one of the researchers of this project (CL).

At the first visit, the research nurse advised participants in the intervention practices to consult their GP to discuss deprescribing their preventive cardiovascular medication. For more details about the factors influencing this decision-making process, refer to an earlier study we performed [17]. When deprescribing was attempted, GPs followed our predefined deprescribing guideline for gradual dose reduction and monitoring of blood pressure and cholesterol levels (Additional file 2). Furthermore, they were advised to follow the recommendations of the Dutch guideline for cardiovascular risk for (re-)initiation of medication (Additional file 2).

No intervention was planned for the GPs, practice nurses and participants in the usual care group.

### Outcome measures

For all participants, we aimed for a follow-up period of 2 years. The primary outcome assessed for non-inferiority was the increase in participants’ predicted 10-year CVD risk in the 2 years after the first visit. Non-inferiority would be declared if the upper limit of the 95% confidence interval (CI) of the difference between the mean increase in CVD risk in the intervention group and the mean increase in CVD risk in the usual care group was below + 2.50 percentage points (on an absolute scale). The non-inferiority margin was set at 2.50 percentage points, because we believed this difference in the increase in the 10-year CVD risk between the intervention group and usual care group was clinically acceptable.

Secondary outcomes assessed for superiority were SBP; diastolic blood pressure (DBP); total cholesterol, HDL-cholesterol and LDL-cholesterol levels; body mass index (BMI; body weight in kilograms/height in metres squared); waist circumference; body weight; smoking behaviour; physical activity; fruit and vegetable intake; and alcohol consumption. These were all assessed at 3 months and 2 years after the first visit. These variables were assessed as outcomes because we hypothesised that the intervention could induce lifestyle changes that could affect these variables. Other secondary outcomes were the negative effects of deprescribing (in the intervention group) and the side effects of antihypertensive and lipid-lowering drugs (in the usual care group) reported by GPs during the trial follow-up, and the incidence of CVD, assessed for superiority 2 years after the first visit.

We performed three post hoc analyses to investigate differences between the intervention group and the usual care group after 2 years of follow-up, using a generalised logistic mixed linear model to assess the relative risk (RR) of (1) having a mean increase in CVD risk > 2.50 percentage points; (2) having hypertension, defined by SBP > 140 mmHg and (3) having hypercholesterolemia, defined by LDL-cholesterol level > 96.5 mg/dl (= 2.5 mmol/l). We did not adjust for the baseline values to calculate RR based on the observed odds ratio (OR) [18].

### Measurements

Participants were visited at baseline (first visit), after 3 months and after 24 months by trained research nurses at the general practice of their GP. During these visits, smoking behaviour was registered, and SBP and DBP were measured twice with a five-minute interval on the arm where SBP at baseline was highest after at least five minutes of seated rest [4] (Omron HEM-907). Additionally, body weight in kilograms (seca 762), height in centimetres (seca 213) and waist circumference in centimetres (seca 201) were measured and registered. The research nurse registered the total cholesterol, HDL-cholesterol and LDL-cholesterol values that local laboratories reported to the general practices.

If research nurses measured a mean SBP > 180 mmHg or registered a total cholesterol level > 308.9 mg/dl (8 mmol/l) or a LDL-cholesterol level > 193.1 mg/dl (5 mmol/l), the participant’s GP was notified.

Two weeks before each visit, participants were asked prospectively to keep a 7-day diary of their alcohol consumption [19] and to complete questionnaires concerning: (1) ethnicity and education level (only at baseline); (2) physical activity (short questionnaire to assess health-enhancing physical activity (SQUASH) [20–22]); and (3) fruit and vegetable intake (standard nutrition questionnaire of Dutch common health services [23]). The research nurse collected and checked the completed questionnaires during the visit.

At 24 months of follow-up, participants in the intervention group were asked to describe their deprescribing status of preventive cardiovascular medication by choosing one of five options: (1) currently not using the medication, (2) currently using fewer or lower doses of the medications, (3) restarted some medications, (4) restarted all medications or (5) never stopped or tried to stop. If participants did not complete the deprescribing status questionnaire, we used the reported negative effects of deprescribing by the GP to search for information about their deprescribing status and the record of the participants’ deprescribing status completed by the research nurse during follow-up.

### Safety

GPs in the intervention group were asked to report the negative effects of deprescribing to the researchers during the trial follow-up, and GPs in the usual care group were asked to report the side effects of antihypertensive and lipid-lowering drugs. Although an assessment of the negative effects of deprescribing in the control group would improve the comparison of the safety profile of the intervention, this was not possible for practical reasons (e.g., to avoid contamination).

The incidence of CVD in participants was determined using the International Classification of Primary Care (ICPC) codes for angina pectoris (K74), acute myocardial infarction (K75), other/chronic ischaemic heart disease (K76), transient ischaemic attack (K89), cerebrovascular accident (K90.03), atherosclerosis (K91), vascular claudication (K92.01) and aortic aneurysm (K99.01), as registered by the GP in the EMR (standard care) during follow-up.

### Economic evaluation

Costs were estimated in the intention-to-treat (ITT) population from a societal perspective at the price level from 2015 [24]. Costs are reported in pounds (based on purchasing power parities of 8 August 2016). Primary-care-specific costs included costs for periodically carried out patient selection (Additional file 3), general practice consultations, antihypertensive and lipid-lowering drug use, and cardiovascular-management-related laboratory measurements; all of these were based on the EMR from the general practices. Total health-care costs also included specialist and physical therapist consultations, use of home care and hospitalisations, all reported by the participants in a cost questionnaire with a 3-month recall period that was administered at 3, 6, 12 and 24 months in the follow-up period (months in between were interpolated). Cost-effectiveness acceptability curves were used to relate the difference in costs to the difference in 2-year quality-adjusted life years (QALYs), as assessed with the Dutch tariff for the EQ-5D-3L questionnaire [25]. Hypothetically, QALYs would be higher in the intervention group compared to the usual care group because of the reduction of the burden of daily medication use and side effects but would be lower because of an increase in the 10-year CVD risk. Acceptability curves show the probability that the intervention has a better net benefit (NB = WTP × QALY – Costs) than the usual care, depending on the willingness to pay (WTP) for one QALY [26].

The economic evaluation was limited to the 2-year trial period, because no reliable information is available to extrapolate the long-term impact on medication use and the balance between side effects, CVD risk and costs in this low-risk population.

### Statistical analysis

For the sample size calculation, we set the expected difference in the increase in the 10-year CVD risk at 1.50 percentage points and the standard deviation at 3.5, and we estimated the number of participating patients per general practice attempting to have their medication deprescribed at 10 (per-protocol or PP population) based on data from a previous study on deprescribing preventive cardiovascular medication in low-CVD-risk patients [12]. We assumed an intraclass correlation coefficient of 0.05, taking into account differences between the participating general practices that could influence study outcomes. The prespecified non-inferiority margin of 2.50 percentage points was based on both statistical reasoning (sample size) and clinical judgement and was set as the maximum allowed upper limit of the 95% CI (one-sided alpha of 5%) of the difference in the increase in the 10-year CVD risk [27]. Assuming that 2/3 of the participants would attempt to have their medication deprescribed, we estimated that 464 × 1.5 = 696 participants from 46 general practices needed to undergo randomisation. Recruitment of general practices was stopped when 46 were included.

During the trial, the proportion of participants attempting to have their medication deprescribed was less than the expected 67% (approximately 55%), while the number of eligible patients per general practice was higher than expected. We, therefore, decided to increase the number of included patients per general practice, allowing us to decrease the planned one-sided alpha from 5% to 2.5%. At the end of the inclusion period, we again had to randomly exclude patients from invitation, because the number of possibly eligible patients per general practice was even higher than anticipated early in the trial (Fig. 1).

The primary outcome was evaluated in the PP population, defined as all patients who were included at the first visit and were allocated to the usual care group and all patients who were included at the first visit in the intervention group who had (attempted to have) their preventive cardiovascular medication stopped based on their self-reported deprescribing status. In non-inferiority trials, an ITT analysis tends to bias towards making the intervention and usual care look similar. Therefore, we chose to evaluate the primary outcome in a PP analysis, as this analysis is more likely to reflect differences between the two treatments [28]. Secondary outcomes were evaluated in the PP population as well. All analyses were repeated for the ITT population. The ITT population is defined as all usual care and intervention group patients who were included at the first visit. Furthermore, we evaluated the primary outcome, SBP, Furthermore, we evaluated the primary outcome, SBP, DBP and LDL-cholesterol levels in the quitters population, defined as all usual care group patients and only those intervention group patients who were able to permanently stop their medication based on their self-reported deprescribing status. The intervention group patients are defined as the ITT intervention group. The intervention group patients who had (attempted to have) their preventive cardiovascular medication stopped are defined as the PP intervention group and the intervention group patients who persisted without cardiovascular medication 2 years after the first visit are defined as the quitters intervention group.

We used multiple imputations to deal with missing values of primary and secondary outcomes and predictors in 15 imputation sets [29]. The following baseline predictors, without any missing values, were used to build the imputation model: allocation group, sex, age, SBP, total cholesterol, HDL-cholesterol, and the utility value of the EQ-5D-3L questionnaire. The clusters were not included as predictors to avoid instability of the model. The imputation model for symmetrically distributed continuous variables was based on linear regression. For skewly distributed continuous variables (skewness statistic > 1 or < -1), predictive mean matching was used. The imputation model for dichotomous variables was based on logistic regression. For missing values of height at baseline, we used the value reported at the end of follow-up and vice versa. For age, we calculated the patient’s age at the median date of the assessments at 24 months of other patients from the same general practice. One intervention group patient, who never attempted to have her preventive cardiovascular medication deprescribed, died of an unknown cause and was left out of our ITT analyses at 24 months.

To compare continuous and binary outcomes, linear mixed and generalised (logistic) mixed linear models were used, respectively, to adjust for cluster randomisation and baseline values of the outcome that was evaluated. Given the low incidence of CVD, the estimation of a cluster effect would be unreliable. Therefore, CVD incidence was analysed with Fisher’s exact test. SPSS Statistics for Windows version 23 was used for all analyses.

## Results

**Fig. 3:**
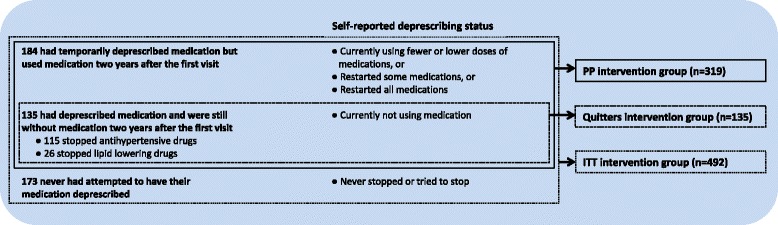
Deprescribing status of preventive cardiovascular medication of the 492 participants in the intervention group. ITT intention-to-treat, PP per-protocol

A total of 1067 participants from 46 general practices (16% of the invited general practices) were included between November 2012 and February 2014 (Fig. 1 and Table 1). The median follow-up period was 23 months (range 17 to 32 months), and the intraclass correlation coefficient for the primary outcome was < 0.01. The ITT intervention group consisted of 492 participants. The PP intervention group consisted of 319 participants (65% of the ITT intervention group) who had (temporarily) deprescribed medication. The quitters intervention group consisted of 135 participants (27% of the ITT intervention group) who persisted without cardiovascular medication 2 years after the first visit (Fig. 3). At baseline, there were some differences between the usual care group, and the PP and ITT intervention groups (Table 1).

**Table 1:** Baseline characteristics of general practices and participants^a^

Characteristic	Usual care group	PP intervention group	ITT intervention group
General practices			
No. of general practices	23	23	23
Years of working experience (as GP) of GP – median (range)	14 (4-36)	21 (1-36)	21 (1-36)
Participants			
No. of participants	575	319	492
Caucasian – no. (%)	543 (94.4)	297 (93.1)	451 (91.7)
Higher education – no. (%)^b^	168 (29.2)	124 (38.9)^c^	180 (36.6)^c^
10-year CVD risk score for inclusion (%)^d^	7.0 (± 5.6)	6.5 (± 4.8)	6.7 (± 4.2)
Cardiovascular risk factors			
10-year CVD risk score (%)^e^	5.1 (± 3.7)	4.7 (± 4.0)	4.9 (± 3.7)
Age (years)	54.9 (± 9.2)	54.5 (± 8.0)	54.5 (± 7.8)
Female – no. (%)	420 (73.0)	229 (71.8)	347 (70.5)
Smokers – no. (%)	66 (11.5)	19 (6.0)^c^	38 (7.7)^c^
Systolic blood pressure (mmHg)	139.8 (± 16.3)	140.4 (± 17.2)	140.9 (± 20.8)
Total cholesterol/HDL-cholesterol ratio	3.7 (± 1.4)	3.7 (± 1.0)	3.8 (± 1.0)
LDL cholesterol (mg/dl)	126.8 (± 55.1)	126.4 (± 38.8)	127.2 (± 42.5)
Medication use at baseline			
Using antihypertensive drugs – no. (%)	479 (83.3)	280 (87.8)	431 (87.6)
Agents acting on the renin-angiotensin system – no. (%)	300 (52.2)	163 (51.1)	276 (56.1)
Diuretics – no. (%)	267 (46.4)	136 (42.6)	216 (43.9)
Beta-blocking agents – no. (%)	154 (26.8)	83 (26.0)	125 (25.4)
Calcium channel blockers – no. (%)	62 (10.8)	37 (11.6)	61 (12.4)
Other antihypertensive drugs – no. (%)	3 (0.5)	1 (0.3)	2 (0.5)
Using antihypertensive drugs from ≥ 2 classes – no. (%)	58 (10.1)	20 (6.3)^c^	44 (8.9)
Using lipid-lowering drugs – no. (%)	163 (28.3)	65 (20.4)^c^	105 (21.3)
HMG CoA reductase inhibitors – no. (%)	162 (28.2)	62 (19.4)^c^	101 (20.5)^c^
Other lipid-lowering drugs – no. (%)	10 (2.0)	8 (2.5)	11 (2.2)
Using both antihypertensive and lipid-lowering drugs – no. (%)	67 (11.7)	27 (8.5)^c^	44 (8.9)^c^

*CVD* cardiovascular disease, *GP* general practitioner, *HDL* high-density lipoprotein, *ITT* intention-to-treat, *LDL* low-density lipoprotein, *PP* per-protocol

^a^Plus or minus values are means ±  standard deviation. All continuous variables were adjusted for cluster randomisation with multilevel linear models

^b^University (of professional education) level

^c^*P* < 0.05 compared to the usual care group

^d^10-year CVD risk score estimated for inclusion with baseline values of age, sex and smoking behaviour, and pre-treatment systolic blood pressure and pre-treatment total cholesterol/HDL-cholesterol ratio as if participants did not use preventive cardiovascular medication

^e^10-year CVD risk score estimated at baseline with baseline values of age, sex, smoking behaviour, systolic blood pressure and total/cholesterol/HDL-cholesterol ratio

Baseline CVD risk in the PP intervention group was 4.7% compared to 5.3% in the 173 intervention group participants who continued their medication or had unknown deprescribing status. The total cholesterol/HDL-cholesterol ratio at baseline was lower in participants who had (temporarily) deprescribed medication. However, there was no difference in age, sex, smoking behaviour, SBP or LDL-cholesterol levels.

For 15% of the participants, at the end of follow-up the levels of SBP, total cholesterol/HDL-cholesterol ratio or smoking behaviour, which are used to determine the primary outcome, were missing and were imputed.

### Primary outcome

**Fig. 4:**
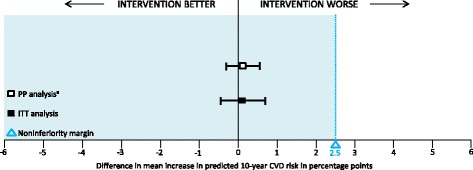
Intention-to-treat and per-protocol analysis of the difference in mean increase in predicted 10-year CVD risk. The error bars depict the 95% CI of the estimated difference in increase of 10-year CVD risk between the usual care and intervention group 2 years after the first visit. ^a^The PP analysis includes only the 319 participants in the intervention group who had (temporarily) deprescibed their preventive cardiovascular medication. CVD cardiovascular disease, CI confidence interval, ITT intention-to-treat, PP per-protocol

The PP analysis showed a 2-year increase in CVD risk in both the intervention group and usual care group, from 4.7% to 6.7% (+ 2.0 percentage points) and from 5.1% to 7.0% (+ 1.9 percentage points), respectively. The mean increase in CVD risk was +0.1 percentage points higher in the deprescribing group, with a 95% CI of -0.3 to 0.6 percentage points, establishing non-inferiority (Fig. 4). The ITT analysis showed similar results. CVD risk increased from 4.9 to 6.9% (+ 2.0 percentage points) in the intervention group, with a mean difference in the increase of 0.1 percentage points (95% CI -0.4 to 0.7).

### Secondary outcomes

**Fig. 5:**
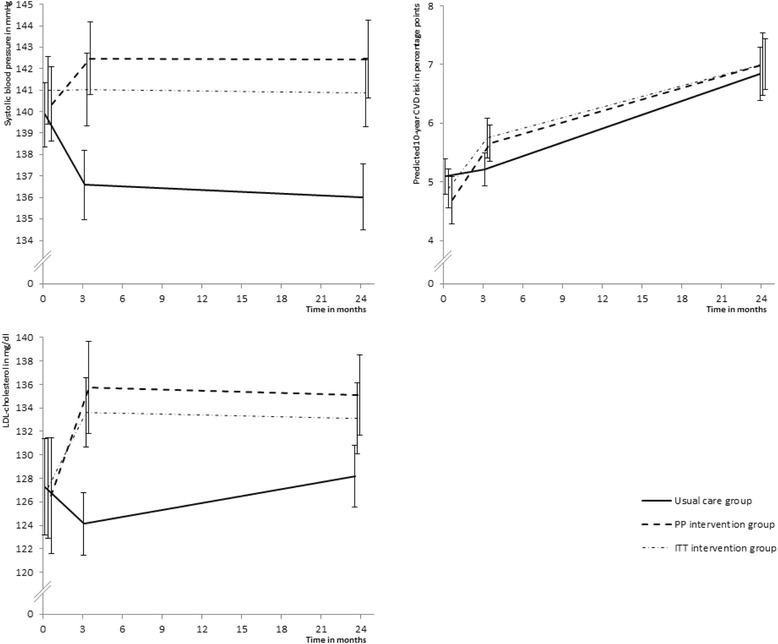
Systolic blood pressure, LDL-cholesterol level and predicted 10-year CVD risk over time in the usual care and intervention groups. Measurements at *t* = 0 were performed at the first visit. Error bars depict the 95% confidence interval of the mean. CVD cardiovascular disease, ITT intention-to-treat, LDL low-density lipoprotein, PP per-protocol

Figure 5 shows SBP, LDL-cholesterol levels and predicted 10-year CVD risk at the first visit, and 3 and 24 months after the first visit. At the end of the follow-up, SBP, DBP, total cholesterol and LDL-cholesterol levels were higher in the PP intervention group compared to the usual care group (all *P* < 0.01, Table 2). Smoking behaviour and BMI were similar in both groups. Physical activity level, fruit and vegetable intake, and alcohol consumption were also similar in both groups. The ITT analysis showed similar results for the secondary outcomes.

**Table 2:** Secondary outcomes after 24 months^a^

Outcome	Usual care group (*n* = 575)	PP intervention group (*n* = 319^b^)		ITT intervention group (*n* = 492^c^)	
	*t* = 24	*t* = 24	*P* value^d^	*t* = 24	*P* value^d^
Systolic blood pressure (mmHg)	136.0 ± 0.8	142.4 ± 0.9	< 0.01	140.9 ± 0.8	< 0.01
Diastolic blood pressure (mmHg)	80.7 ± 0.5	84.8 ± 0.6	< 0.01	84.2 ± 0.5	< 0.01
Total cholesterol/HDL-cholesterol ratio	3.83 ± 0.04	3.89 ± 0.05	0.22	3.90 ± 0.05	0.35
Total cholesterol (mg/dl)^e^	210.0 ± 1.4	217.2 ± 1.8	< 0.01	214.1 ± 1.6	0.05
HDL-cholesterol (mg/dl)^e^	58.4 ± 0.5	59.1 ± 0.6	0.75	58.3 ± 0.5	0.84
LDL-cholesterol (mg/dl)^e^	128.2 ± 1.3	135.1 ± 1.7	< 0.01	133.1 ± 1.5	0.01
Smokers – no. (%)^f^	59 (10.3)	18 (5.6)	> 0.31	35 (7.1)	> 0.25
Body mass index (weigh tin kg/height in metres squared)	28.0 ± 0.1	27.6 ± 0.1	0.26	27.9 ± 0.1	0.57
Body weight (kg)	81.5 ± 0.3	80.4 ± 0.3	0.18	81.1 ± 0.3	0.35
Waist circumference (cm)	96.1 ± 0.4	96.2 ± 0.5	0.54	96.5 ± 0.4	0.53
Physical activity level (minutes per day)^g^	137 ± 5	127 ± 6	0.18	130 ± 6	0.36
Fruit and vegetable consumption (grams per day)	329 ± 5	335 ± 7	0.41	333 ± 6	0.62
Alcohol consumption (glasses per day)	0.97 ± 0.05	0.90 ± 0.06	0.29	0.87 ± 0.05	0.10

*HDL* high-density lipoprotein, *ITT* intention-to-treat, *LDL* low-density lipoprotein, *PP* per-protocol

^a^Plus or minus values are means ± standard error from linear mixed models

^b^Only participants who had (temporarily) deprescribed their preventive cardiovascular medication were analysed in the PP intervention group

^c^One participant who died of unknown cause during the follow-up without having attempted to have her preventive cardiovascular medication deprescribed was left out in the analyses at 24 months in the ITT population of the intervention group

^d^Compared to the usual care group at 24 months

^e^To change value to mmol/l, multiply by 38.61033861

^f^Using a generalised logistic mixed linear model adjusting for cluster randomisation did not result in a pooled estimate. Therefore, we calculated estimates for the 15 imputation sets and reported the lowest *P* value in this table

^g^For patients < 55 years old, only activities with a metabolic equivalent (MET) score ≥ 4 kcal/kg/hour executed ≥ 60 minutes on one or more days were taken into account to assess physical activity level [22]. For patients ≥ 55 years old, only activities with a MET-score ≥ 3 kcal/kg/hour executed ≥ 30 minutes on one or more days were taken into account to assess physical activity level [22]

### Cardiovascular events and other negative effects

In the usual care group, eight participants developed CVD during follow-up and zero developed CVD in the PP intervention group (*P* = 0.03). In the ITT intervention group, two participants developed CVD (*P* = 0.12 compared to the usual care group). CVD incidence could not be identified in 61 participants because of withdrawn informed consent (29 in the usual care group, 5 in the PP intervention group and 32 in the ITT intervention group).

In the PP intervention group, GPs reported 76 negative effects because of deprescribing in 42 of 319 participants (13.2%) (Table 3). Antihypertensive and/or lipid-lowering drugs were restarted in 34 of these 42 participants. GPs in the usual care group reported no side effects of antihypertensive or lipid-lowering drugs during follow-up.

**Table 3:** Negative effects of deprescribing reported to the researchers by GPs in the intervention group^a^

Negative effects	Participants (*n* = 42)	Only restarted participants (*n* = 34)
	Times reported	Times reported
Hypertension or increased blood pressure	24	21
Headache or migraine	18	11
Nervous or stressed feeling	7	5
Palpitations	7	5
Ankle oedema or fluid buildup	4	3
Hypercholesterolemia	4	4
Pressure sensation on chest	2	2
Dizziness	2	2
Not feeling well	2	2
Tachycardia	1	1
Systolic cardiac souffle	1	1
Dyspnoea	1	1
Fatigue	1	1
Nausea	1	1
Hot flushes	1	1
Total	76	61

*GP* general practitioner

^a^GPs in the usual care group did not report any side effect of antihypertensive or lipid-lowering drugs to the researchers during follow-up

### Quitters

An analysis of 135 participants who were still not taking medication 2 years after the first visit (Fig. 3), showed a 2-year increase in CVD risk from 4.3% to 6.6% (+ 2.3 percentage points). This increase was a +0.4 percentage points higher compared to the usual care group, with a 95% CI of -0.3 to 1.1 percentage points, establishing non-inferiority. Two years after the first visit, the difference in SBP between the quitters intervention group and the usual care group was 10 mmHg (146 vs. 136 mmHg, respectively). The difference in DBP was 7 mmHg (87 vs. 80 mmHg, respectively) and the difference in LDL-cholesterol was 13 mg/dl (141 vs. 128 mg/dl, respectively). All were *P* < 0.01.

The difference in SBP between the 115 participants who had their antihypertensive drugs deprescribed (Fig. 3) compared to the 479 participants using antihypertensive drugs at baseline in the usual care group was 13 mmHg 2 years after the first visit (149 vs. 136 mmHg, respectively, *P* < 0.01). The difference in LDL-cholesterol of the 26 participants who had their lipid-lowering drugs deprescribed (Fig. 3) compared to the 163 participants using lipid-lowering drugs at baseline in the usual care group was 56 mg/dl (178 vs. 122 mg/dl, respectively, *P* < 0.01).

### Individual follow-up

The RR of having a mean increase in CVD risk > 2.5 percentage points after 2 years of follow-up for the PP intervention group vs. the usual care group was 1.29 (95% CI 1.01 to 1.61, based on a baseline risk of 0.222 and an OR of 1.40). The RR of having SBP > 140 mmHg and the RR of having a LDL-cholesterol level > 96.5 mg/dl for the PP intervention group vs. the usual care group was 1.41 (95% CI 1.18 to 1.64, based on a baseline risk of 0.372 and an OR of 1.87) and 1.10 (95% CI 1.04 to 1.15, based on a baseline risk of 0.807 and an OR of 1.96), respectively. The ITT analysis showed similar results for having SBP > 140 mmHg and having LDL-cholesterol level > 96.5 mg/dl, as the RR and 95% CI in the ITT analysis were comparable to the RR and 95% CI resulting from the PP analysis. In the ITT analysis, the RR of having a mean increase in CVD risk was > 2.5 percentage points for the PP intervention group vs. the usual care group was 1.21 (95% CI 0.97 to 1.49).

### Economic evaluation

In the first year, intervention costs and GP consultation costs were higher in the ITT intervention group by £86 per participant (Additional file 3: Table S3, *P* < 0.01). In both years, medication costs were lower in the ITT intervention group by £28 (*P* < 0.01). Total 2-year health-care costs and primary-care costs did not differ between the two groups (*P* = 1.00 and *P* = 0.19, respectively). In addition, no difference was found in QALYs (*P* = 0.45) (Additional file 3: Table S3). Whether an attempt to deprescribe preventive cardiovascular medication is cost-effective depends on how much one is willing to pay for 1 QALY. Figure S2 in Additional file 3 shows the probability that an attempt to deprescribe preventive cardiovascular medication in general practice is cost-effective compared with usual care. An attempt to deprescribe preventive cardiovascular medication is 70% to 80% likely to be cost-effective for a WTP between £20,000 and £30,000.

## Discussion

The ECSTATIC study revealed that an attempt to deprescribe preventive cardiovascular medication in patients in general practice with predicted low 10-year CVD risk was safe in the short term compared to usual care based on a minimal difference in the increase in predicted 10-year CVD risk. After 2 years of follow-up, the mean blood pressure was 6 mmHg higher, and the total cholesterol and LDL-cholesterol levels were both on average 7 mg/dl higher compared to usual care in the intervention group. The risk of having hypertension after 2 years of follow-up was approximately 20% to 60% higher in the intervention group and the risk of having hypercholesterolemia was approximately 5% to 15% compared to the usual care group. Only 27% of participants persisted without medication 2 years after the first visit. In the intervention group, 1-year primary-care costs were higher, but 2-year primary-care costs and total health-care costs were similar and there was no difference in QALYs.

Based on our findings, an attempt to deprescribe preventive cardiovascular medication in low-CVD-risk patients is safe when blood pressure and cholesterol levels are monitored after stopping, but it does not improve the quality of life or reduce health-care costs.

### Strengths and weaknesses of this study

Study strengths include the large sample of general practices and patients, and the pragmatic trial design. Both of these reflect the results of implementing such an intervention in daily practice.

The ECSTATIC study was not designed to answer questions about efficacy, but was designed as a pragmatic trial to answer the question of whether a structured deprescribing strategy in low-CVD-risk patients is (cost-)effective when implemented in general practice [30]. The pragmatic choice to leave the decision to deprescribe to the patient and their GP and the choice to use an active control group may have resulted in an underestimation of the effect of the intervention on CVD risk, blood pressure and cholesterol levels [31]. A PP analysis gave information on the potential effects of an attempt to deprescribe preventive cardiovascular medication.

The differences at baseline between the intervention group and the usual care group may be the consequence of the different invitation letters that both groups received. We minimised the effect of these differences by correcting all analyses (except for the post hoc analyses) for baseline values.

Our choice to include participants in the trial based on their predicted 10-year CVD risk was practice-driven. Although current debate questions the use of population-based prediction models for drug treatment in individuals, these models seem to predict individual CVD risk better in low risk than in high risk populations [32, 33]. The predicted 10-year CVD risk score is designed to assess risk while off treatment. However, the predictions of this risk assessment tool are partly based on cohorts of patients using cardiovascular medication, justifying its use in our patient population [34]. Additionally, our choice to include participants based on their 10-year CVD risk was based on the best available evidence as aggregated in the current Dutch guideline for Cardiovascular Risk Management. The long-term incidence of CVD would have been the optimal primary outcome measure for our trial. However, time and budgetary restrictions prevented us from using this endpoint. We would encourage future studies to compare a deprescribing strategy with usual care in low-CVD-risk patients based on long-term incidence of CVD.

For 15% of the participants, we had to impute for missing data to be able to analyse the primary outcome. This number of missing data points may lead to less reliable results. However, we used rigorous imputation methods to ensure the validity of our data and the precision of our results [29]. It was hard to verify based on the EMRs whether a medication was stopped after 2 years of follow-up or whether it was just not yet prescribed again. Therefore, self-reported deprescribing status seemed more reliable. The self-reported deprescribing status may have led to incorrect allocations to the ITT intervention group, PP intervention group and the quitters intervention group. However, SBP and LDL-cholesterol levels in the PP intervention group and the quitters intervention group were higher than in the ITT intervention group, suggesting that the allocation was quite reliable.

Because adequate registration of cardiovascular events in the EMRs is usual practice and its extraction based on ICPC codes was protocolised, we believe the lack of blinding did not prevent objective registration and collection of events.

### Comparison with other studies and interpretation

The ECSTATIC study adds to the body of knowledge concerning preventive cardiovascular drug treatment as the primary prevention of CVD because of its pragmatic design. It was carried out in a primary-care population with low average CVD risk.

The preventive effects of antihypertensive and lipid-lowering drugs in low-CVD-risk populations are less than in intermediate-risk and high-risk populations [35–39]. The HOPE-3 investigators found that treatment with 16 mg of candesartan and 12.5 mg of hydrochlorothiazide per day by intermediate-risk patients did not result in a significantly lower risk of major cardiovascular events compared to a placebo [37]. Antihypertensive therapy reduced CVD risk only in intermediate-risk patients with higher baseline SBP (> 143.5 mmHg) [37]. Furthermore, the meta-analysis of the Cholesterol Treatment Trialist collaborators found that a statin-induced LDL-cholesterol reduction of 1 mmol/l (38.6 mg/dl) in patients without vascular disease with a 5-year major vascular event risk < 5%, did lower the rate ratio of vascular events, though not the rate ratio of vascular death [35]. The findings of these studies are consistent with the similar and low incidence of CVD (although underpowered) in the usual care group and intervention group and the non-inferiority of an attempt to deprescribe in the ECSTATIC population (which had a mean SBP of 140 mmHg at baseline, and a mean 10-year CVD risk of 5%).

With a mean 10-year CVD risk of 5%, the ECSTATIC population has lower risk compared to populations in other trials. Based on their reports of baseline characteristics, the study populations of recent trials, such as the JUPITER Study (approximately 15% 10-year CVD risk), the HOPE-3 trial (approximately 17% 10-year CVD risk) and the SPRINT trial (approximately 24% 10-year CVD risk), have higher risks at baseline, predominantly because of the higher ages and fewer female participants [37, 40–43]. The findings from these trials cannot, therefore, be directly compared with the ECSTATIC population. With a mean 10-year CVD risk of approximately 6%, mean age of 58.3 years and inclusion of 68% women, the total Asian population in the MEGA Study is most comparable to the ECSTATIC population [44]. The MEGA Study showed that statins reduce the relative risk of coronary heart disease in a subgroup of patients with LDL-cholesterol levels > 4.01 mmol/l (155 mg/dl) [44]. This suggests that the 26 ECSTATIC participants who had their lipid-lowering drugs deprescribed 2 years after the first visit, with a mean LDL-cholesterol level of 178 mg/dl, may benefit from the preventive effects of statin use. However, other evidence suggests that the increase in total life expectancy and CVD-free life expectancy may be too small to justify long-term statin use at all, especially in an ageing population [45].

It is remarkable that 35% of the participants in the intervention group of the ECSTATIC study did not attempt to have their medication deprescribed. Based on the findings of two of our previous studies, possible reasons for not doing an attempt are, for example, fear of the consequences of deprescribing, fear of cardiovascular events, the lack of negative effects of the medication participants experienced, or the GPs’ doubts about deprescribing [17, 46]. Furthermore, only 27% of the participants in the intervention group persisted in quitting, while 65% of the participants did attempt to have their medication deprescribed. Reasons for restarting medication were scarcely reported by GPs, as reasons were reported in only 34 restarted participants (18% of all restarted participants). However, hypertension, headaches, nervousness and stress, or palpitations were most frequently mentioned by GPs as reasons for restarting medication.

## Conclusions

The results of the ECSTATIC study show that an attempt to deprescribe preventive cardiovascular medication in patients with predicted low CVD risk is safe in the short term, but does not necessarily improve quality of life or reduce health-care costs. Moreover, less than one third of participants persisted without cardiovascular medication after 2 years of follow-up. Therefore, we do not recommend implementation of a structured deprescribing strategy for all patients with low CVD risk in general practice as was implemented in the intervention group of the ECSTATIC study.

However, an attempt to deprescribe may be considered in low-CVD-risk patients, e.g., during their routine (yearly) cardiovascular check-up and as the result of a shared decision between a doctor and their patient. In an earlier study, we found that low-CVD-risk patients and their GPs may doubt the appropriateness of medication use, fear side effects, dislike medication use and consider alternative prevention options [17]. Although an attempt to deprescribe medication increases the risk of developing hypertension by approximately 20% to 60% and the risk of developing hypercholesterolemia by approximately 5% to 15%, the balance of the risks of (future) side effects and benefits for individual patients (e.g., no burden of daily medication use), together with patients’ preferences, may drift in the direction of an individual attempt to deprescribe medication. When an attempt to deprescribe preventive cardiovascular medication in low-CVD-risk patients is made, it is important to monitor blood pressure and cholesterol levels, especially in the first 3 months after withdrawal, and to assess whether drug treatment should be re-initiated. Combining deprescribing with a lifestyle intervention could possibly restrict increases in blood pressure and cholesterol levels and lower CVD risk [47–49].

In conclusion, a structured deprescribing strategy for all patients with low CVD risk in general practice is not recommended because of its low adherence (27% persistent quitters) and low gains in quality of life, but an attempt to deprescribe for those willing to is safe in the short term when blood pressure and cholesterol levels are monitored after stopping and can therefore, be considered in low-CVD-risk patients during routine visits.

## Additional files

Additional file 1: Inclusion and exclusion criteria of the Evaluating Cessation of Statins and Antihypertensive Treatment in Primary Care Trial, as approved by the Medical Ethics Committee of Leiden University Medical Centre. (DOCX 22 kb)

Additional file 2: Deprescribing guideline. **Table S1.** Examples of dose-lowering schemes. (DOCX 18 kb)

Additional file 3: Cost-effectiveness analysis. **Table S2.** Costs (in £) for the preparation of the intervention, per selected patient. **Table S3.** Costs (in £) and QALYs per patient in usual care group and intervention group. **Figure S1.** EuroQol Utility at *t* = 0, *t* = 3, *t* = 6, *t* = 12 and *t* = 24 in the usual care group and the intention. **Figure S2.** Cost-effectiveness acceptability curve showing the probability that an attempt to deprescribe preventive cardiovascular medication is cost-effective compared to usual care. (DOCX 66 kb)

## Abbreviations

BMI: Body mass index
CI: confidence interval
CVD: Cardiovascular disease
DBP: Diastolic blood pressure
ECSTATIC: Evaluation Cessation of STatins and Antihypertensive Treatment In primary Care
EMR: Electronic medical record
EQ-5D-3L: EuroQol five-dimension questionnaire
FH: Familial hypercholesterolemia
GP: General practitioner
HDL: High-density lipoprotein
ICPC: International Classification of Primary Care
ITT: Intention-to-treat
LDL: Low-density liposprotein
MET: metabolic equivalent
OR: Odds ratio
PP: Per-protocol
QALY: Quality-adjusted life year
RR: Relative risk
SBP: Systolic blood pressure
SQUASH: Short questionnaire to assess health-enhancing physical activity
WTP: Willingness to pay

## References

[1] GBD 2013 Mortality and Causes of Death Collaborators: Global, regional, and national age-sex specific all-cause and cause-specific mortality for 240 causes of death, 1990-2013: A systematic analysis for the Global Burden of Disease Study 2013. Lancet. 2015;385:117–71. 10.1016/S0140-6736(14)61682-2 PMC4340604 25530442

[2] Vaartjes I, van Dis I, Visseren FLI, Bots ML (2011). Hart- en vaatziekten in Nederland 2010. Cijfers over leefstijl- en risicofactoren, ziekte en sterfte.

[3] Piepoli MF, Hoes AW, Agewall S, Albus C, Brotons C, Catapano AL, et al. European Guidelines on cardiovascular disease prevention in clinical practice. Eur Heart J. 2016;37:2315–81.

[4] Wiersma T, Smulders YM, Stehouwer CD, Konings KT, Lanphen J (2012). Summary of the multidisciplinary guideline on cardiovascular risk management (revision 2011). Ned Tijdschr Geneeskd.

[5] Gohlke H, Gielen S, König W, Schuler G, Rauch B, Sonntag F. ESC Pocket Guidelines Prävention von Herz-Kreislauf-Erkrankungen. Düsseldorf: Deutsche Gesellschaft für Kardiologie - Herz- un Kreislauf Forschung eV (DGK); 2012. 31 p. http://leitlinien.dgk.org/files/PL_Pr%C3%A4vention_Internet_13.pdf. Accessed 25 Sep 2017.

[6] National Heart Foundation of Australia (National Blood Pressure and Vascular Disease Advisory Committee) (2010). Guide to management of hypertension 2008. Updated December 2010.

[7] Cardiovascular Disease Risk Assessment Steering Group (2013). New Zealand primary care handbook 2012.

[8] Walma E, Thomas S, Prins A, Grundmeijer H, Van der Laan J, Wiersma T (2003). NHG-standaard hypertensie. Huisarts en Wetenschap.

[9] Thomas S, van der Weijden T, van Drenth B, Haverkort A, Hooi J, van der Laan J (1999). NHG-standaard cholesterol.

[10] Ärztliche Zentralstelle Qualitätssicherung. Leitlinien-Clearing-Bericht “Hypertonie”. Köln: Ärztliche Zentralstelle Qualitätssicherung; 2000. http://www.leitlinien.de/mdb/edocs/pdf/schriftenreihe/schriftenreihe5.pdf. Accessed 25 Sep 2017.

[11] Luymes CH, de Ruijter W, Poortvliet RK, Putter H, van Duijn HJ, Numans ME (2015). Change in calculated cardiovascular risk due to guideline revision: a cross-sectional study in the Netherlands. Eur J Gen Pract.

[12] van Duijn HJ, Belo JN, Blom JW, Velberg ID, Assendelft WJ (2011). Revised guidelines for cardiovascular risk management – time to stop medication? a practice-based intervention study. Br J Gen Pract.

[13] Cahir C, Bennett K, Teljeur C, Fahey T (2014). Potentially inappropriate prescribing and adverse health outcomes in community dwelling older patients. Br J Clin Pharmacol.

[14] Beers MH, Ouslander JG, Rollingher I, Reuben DB, Brooks J, Beck JC (1991). Explicit criteria for determining inappropriate medication use in nursing home residents. Arch Intern Med.

[15] Anderson K, Stowasser D, Freeman C, Scott I (2014). Prescriber barriers and enablers to minimising potentially inappropriate medications in adults: a systematic review and thematic synthesis. BMJ Open.

[16] Schellings R, Kessels AG, ter Riet G, Sturmans F, Widdershoven GA, Knottnerus JA (2009). Indications and requirements for the use of prerandomization. J Clin Epidemiol.

[17] Luymes CH, van der Kleij RM, Poortvliet RK, de Ruijter W, Reis R, Numans ME (2016). Deprescribing potentially inappropriate preventive cardiovascular medication: barriers and enablers for patients and general practitioners. Ann Pharmacother.

[18] Grant RL (2014). Converting an odds ratio to a range of plausible relative risks for better communication of research findings. BMJ.

[19] Lemmens P, Tan ES, Knibbe RA (1992). Measuring quantity and frequency of drinking in a general population survey: a comparison of five indices. J Stud Alcohol.

[20] Wendel-Vos GC, Schuit AJ, Saris WH, Kromhout D (2003). Reproducibility and relative validity of the short questionnaire to assess health-enhancing physical activity. J Clin Epidemiol.

[21] Ainsworth BE, Haskell WL, Leon AS, Jacobs DR, Montoye HJ, Sallis JF (1993). Compendium of physical activities: classification of energy costs of human physical activities. Med Sci Sports Exerc.

[22] Kemper H, Ooijendijk W, Stiggelbout M (2000). FORUM-consensus over de nederlandse norm voor gezond bewegen. TSG-Tijdschrift voor Gezondheidswetenschappen.

[23] Brink CLvd, Ocké MC, Houben AW, Nierop Pv, Droomers M. Validering van standaardvraagstelling voeding voor Lokale en Nationale Monitor Volksgezondheid, RIVM rapport 260854008/2005. 2005. http://www.rivm.nl/dsresource?objectid=e370650a-dd54-4ccc-b4f1-1f26c0494215&type=org&disposition=inline. Accessed 25 Sep 2017.

[24] Nederland Z (2015). Richtlijn voor het uitvoeren van economische evaluaties in de gezondheidszorg.

[25] Lamers LM, McDonnell J, Stalmeier PF, Krabbe PF, Busschbach JJ (2006). The Dutch tariff: results and arguments for an effective design for national EQ‐5D valuation studies. Health Econ.

[26] Zethraeus N, Johannesson M, Jönsson B, Löthgren M, Tambour M (2003). Advantages of using the net-benefit approach for analysing uncertainty in economic evaluation studies. Pharmacoeconomics.

[27] ICH Steering Committee. Harmonised Tripartite Guideline: Choice of Control Group and Related Issues in Clinical Trials (E10). Geneva, Switzerland: International Conference on Harmonisation of Technical Requirements for Registration of Pharmaceuticals for Human Use; 2000.https://www.ich.org/fileadmin/Public_Web_Site/ICH_Products/Guidelines/Efficacy/E10/Step4/E10_Guideline.pdf.

[28] D'Agostino RB, Massaro JM, Sullivan LM (2003). Non‐inferiority trials: design concepts and issues – the encounters of academic consultants in statistics. Stat Med.

[29] Bodner TE (2008). What improves with increased missing data imputations?. Struct Equ Model.

[30] Thabane L, Kaczorowski J, Dolovich L, Chambers LW, Mbuagbaw L (2015). Reducing the confusion and controversies around pragmatic trials: using the cardiovascular health awareness program (CHAP) trial as an illustrative example. Trials.

[31] Chapman RH, Benner JS, Petrilla AA, Tierce JC, Collins SR, Battleman DS (2005). Predictors of adherence with antihypertensive and lipid-lowering therapy. Arch Intern Med.

[32] van Staa T-P, Gulliford M, Ng ES-W, Goldacre B, Smeeth L (2014). Prediction of cardiovascular risk using Framingham, ASSIGN and QRISK2: how well do they predict individual rather than population risk?. PLoS One.

[33] Björnson E, Borén J, Mardinoglu A. Personalized cardiovascular disease prediction and treatment – a review of existing strategies and novel systems medicine tools. Front Physiol. 2016;7:2. 10.3389/fphys.2016.00002 PMC4726746 26858650

[34] Liew SM, Doust J, Glasziou P (2011). Cardiovascular risk scores do not account for the effect of treatment: a review. Heart.

[35] Cholesterol Treatment Trialists' (CTT) Collaborators (2012). The effects of lowering LDL cholesterol with statin therapy in people at low risk of vascular disease: meta-analysis of individual data from 27 randomised trials. Lancet.

[36.Z] Taylor F, Huffman MD, Macedo AF, Moore TH, Burke M, Davey Smith G, et al. Statins for the primary prevention of cardiovascular disease. Cochrane Database Syst Rev. 2013;1:CD004816. 10.1002/14651858.CD004816.pub5 PMC6481400 23440795

[37] Lonn EM, Bosch J, López-Jaramillo P, Zhu J, Liu L, Pais P (2016). Blood-pressure lowering in intermediate-risk persons without cardiovascular disease. N Engl J Med.

[38] Ettehad D, Emdin CA, Kiran A, Anderson SG, Callender T, Emberson J (2016). Blood pressure lowering for prevention of cardiovascular disease and death: a systematic review and meta-analysis. Lancet.

[39] Ray KK, Seshasai SRK, Erqou S, Sever P, Jukema JW, Ford I (2010). Statins and all-cause mortality in high-risk primary prevention: a meta-analysis of 11 randomized controlled trials involving 65 229 participants. Arch Intern Med.

[40] Ridker PM, Danielson E, Fonseca F, Genest J, Gotto AM, Kastelein J (2008). Rosuvastatin to prevent vascular events in men and women with elevated C-reactive protein. N Engl J Med.

[41] Yusuf S, Lonn E, Pais P, Bosch J, López-Jaramillo P, Zhu J (2016). Blood-pressure and cholesterol lowering in persons without cardiovascular disease. N Engl J Med.

[42] Yusuf S, Bosch J, Dagenais G, Zhu J, Xavier D, Liu L (2016). Cholesterol lowering in intermediate-risk persons without cardiovascular disease. N Engl J Med.

[43] The SPRINT Research Group (2015). A randomized trial of intensive versus standard blood-pressure control. N Engl J Med.

[44] Nakamura H, Arakawa K, Itakura H, Kitabatake A, Goto Y, Toyota T (2006). Primary prevention of cardiovascular disease with pravastatin in Japan (MEGA Study): a prospective randomised controlled trial. Lancet.

[45] Ferket BS, van Kempen BJ, Heeringa J, Spronk S, Fleischmann KE, Nijhuis RL (2012). Personalized prediction of lifetime benefits with statin therapy for asymptomatic individuals: a modeling study. PLoS Med.

[46] Luymes CH, Boelhouwer NJ, Poortvliet RK, de Ruijter W, Reis R, Numans ME (2017). Understanding deprescribing of preventive cardiovascular medication: a Q-methodology study in patients. Patient Prefer Adherence.

[47] Hartley L, Igbinedion E, Holmes J, Flowers N, Thorogood M, Clarke A, et al. Increased consumption of fruit and vegetables for the primary prevention of cardiovascular diseases. Cochrane Database Syst Rev. 2013;6:CD009874. 10.1002/14651858.CD009874.pub2 PMC6464871 23736950

[48] Hooper L, Summerbell CD, Thompson R, Sills D, Roberts FG, Moore HJ, et al. Reduced or modified dietary fat for preventing cardiovascular disease. Cochrane Database Syst Rev. 2012;5:CD002137. 10.1002/14651858.CD002137.pub3 PMC6486029 22592684

[49] Shaw KA, Gennat HC, O'Rourke P, Del Mar C. Exercise for overweight or obesity. Cochrane Database Syst Rev. 2006;4:CD003817. 10.1002/14651858.CD003817.pub3 PMC9017288 17054187

