# Supplementary material for: Deprescribing preventive cardiovascular medication in patients with predicted low cardiovascular disease risk in general practice – the ECSTATIC study: a cluster randomised non-inferiority trial
Source: BMC Med. 2018 Jan 11;16:5. doi: 10.1186/s12916-017-0988-0 (PMC5763574; doi:10.1186/s12916-017-0988-0)
Supplement: Supplementary file 1 — Inclusion and exclusion criteria of the Evaluating Cessation of Statins and Antihypertensive Treatment in Primary Care Trial, as approved by the Medical Ethics Committee of Leiden University Medical Centre. (DOCX 22 kb) [file 12916_2017_988_MOESM1_ESM.docx]

**Additional file 1. Inclusion and exclusion criteria of the Evaluating Cessation of STatins and Antihypertensive Treatment In primary Care trial, as approved by the Medical Ethics Committee of the Leiden University Medical Center

Inclusion criteria**

Patients in general practice are included in the study if they meet the following criteria:

- Age 40 to 70 years;
- Prescription and use of antihypertensive medication and/or lipid-lowering drugs for hypertension and/or hypercholesterolemia during the last 12 months (using ATC codes: C02, C03, C07, C08, C09, C10).

**Exclusion criteria**

Patients with the following criteria are excluded:

- Cardiovascular disease (ICPC codes: K74, K75, K76, K89, K90.03, K91, K92.01 and K99.01)^1^;
- Use of platelet aggregation inhibitors (heparin excluded) (ATC code: B01AC);
- Use of antihypertensive medication for another reason than prevention of CVD;
- Familial hypercholesterolemia/lipidemia (ICPC code: T93.04)^a^;
- Patients with a current SBP^b^ >180 mmHg, or a SBP >180 mmHg before the start of medication;
- Patients with a current TC/HDL ratio >8, or a TC/HDL ratio >8 before the start of medication;
- Patients with a 10-year CVD risk >16%^c^
- 10-year CVD risk of 10-16% based on the 2011 risk table, in combination with **at least one** **additional major risk-increasing factor**:
  - Family history with ≥2 first degree family members with CVD <65 years or ≥1 first degree family member with CVD <60 years;
  - Physical activity: sedentary lifestyle;
  - Obesity: BMI >35 kg/m^3^ ;
  - Kidney function: eGFR <30 ml/min/1.73m^2^;
  - DM and poor metabolic control;
  - DM and (micro)albuminuria;
  - DM and microvascular complications;
  - RA with high disease activity.
- 10-year CVD risk of 10-16%, based on the 2011 risk table, in combination with **two or more** **additional minor risk-increasing factors**:
  - Family history: 1 first degree family member with CVD <65 years;
  - Physical activity: <30 min/day ≤5 days per week (but not sedentary);
  - Obesity: BMI >30-35 kg/m^3^ (or waist circumference >80 cm in women, >94 cm in men;
  - Kidney function: eGFR <65 years 30-60 ml/min/1.73m^2^, eGFR ≥65 years 30-45 ml/min/1.73m^2^.
- 10-year CVD risk of 10-16%, based on the 2011 risk table, in combination with **one** **additional minor risk-increasing factor**:
  - Family history: 1 first degree family member with CVD <65 years;
  - Physical activity: <30 min/day ≤5 days per week (but not sedentary);
  - Obesity: BMI >30-35 kg/m^3^ (or waist circumference >80 cm in women, >94 cm in men;
  - Kidney function: eGFR <65 years 30-60 ml/min/1.73m^2^, eGFR ≥65 years 30-45 ml/min/1.73m^2^;

**AND**

- - SBP >140 mmHg and/or LDL >2.5 mmol/L.
- Patients <50 years with a repeatedly measured SBP >160 mmHg who do not reach their target SBP with help of lifestyle adjustments after 3 months.^d^

Abbreviations: SBP, systolic blood pressure; TC, total cholesterol; HDL, HDL-cholesterol; CVD, cardiovascular disease; BMI, body mass index; eGFR, estimated glomerular filtration rate; DM, diabetes mellitus; RA, rheumatoid arthritis; LDL, LDL-cholesterol.
^a^ URL: http://www.kith.no/upload/2705/icpc-2-english.pdf

^b^ SBP was measured once at both arms, and twice at the arm with highest SBP at the first measurement after at least 5 minutes sitting rest; mean SBP of the two measurements at the same arm was noted as current SBP and SBP at baseline.
^c^ Patients with a 10-year CVD risk >16.99% (≥17%) were excluded.
^d^ This exclusion criterion appeared to be infeasible in practice.

**Calculation of pre-treatment 10-year CVD risk**Pre-treatment CVD risk was based on current age, sex, and smoking behaviour (smoking yes/no), in combination with reported pre-treatment systolic blood pressure (SBP) and total cholesterol/HDL-cholesterol ratio levels in general practice EMRs. If these values were not available up to one year before the start of drug treatment, pre-treatment SBP was conservatively estimated at 180 mmHg; to estimate the pre-treatment total cholesterol ratio, 72 mg/dl (2.0 mmol/l) was added to the current total cholesterol level and 4 mg/dl (0.1 mmol/l) was subtracted from the current HDL level. In case patients were using only antihypertensive drugs, the current total cholesterol/HDL-cholesterol ratio was used to estimate the pre-treatment CVD risk; in case patients were using only lipid-lowering drugs, current SBP was used to estimate pre-treatment CVD risk. The algorithm for the CVD risk calculation can be requested from the Dutch College of General Practitioners (Nederlands Huisartsen Genootschap, Utrecht, the Netherlands). Current SBP was measured with an automated sphygmomanometer (Omron HEM-907) once on both arms and twice on the arm with highest SBP at the first measurement, with an interval of five minutes after at least five minutes of seated rest. The mean SBP of the two measurements on the same arm was used as the current SBP to estimate the pre-treatment CVD risk. Local laboratories measured current levels of total cholesterol, HDL-cholesterol, and LDL- cholesterol.
We used an SBP of 120 mmHg for a mean SBP <120 mmHg to estimate CVD risk; an SBP of 180 mmHg, for a mean SBP >180 mmHg; a total cholesterol/HDL-cholesterol ratio of 4, when total cholesterol/HDL-cholesterol ratio <4; and a total cholesterol/HDL-cholesterol ratio of 8, when total cholesterol/HDL-cholesterol ratio was >8.
In addition, for patients with intermediate pre-treatment CVD risk, to determine whether a patient met the inclusion criteria, current glomerular filtration rate (estimated using the Modification of Diet in Renal Disease Study equation measured by local laboratories) was used, together with current body mass index (BMI), physical activity level, and family history of CVD in accordance with the Dutch guideline.[^1^](#_ENREF_1)

REFERENCES

1. Wiersma T, Smulders YM, Stehouwer CD, Konings KT, Lanphen J. [Summary of the multidisciplinary guideline on cardiovascular risk management (revision 2011)]. *Ned Tijdschr Geneeskd* 2012;156:A5104.
