# Supplementary material for: Deprescribing preventive cardiovascular medication in patients with predicted low cardiovascular disease risk in general practice – the ECSTATIC study: a cluster randomised non-inferiority trial
Source: BMC Med. 2018 Jan 11;16:5. doi: 10.1186/s12916-017-0988-0 (PMC5763574; doi:10.1186/s12916-017-0988-0)
Supplement: Supplementary file 2 — Deprescribing guideline. Table S1. Examples of dose-lowering schemes. (DOCX 18 kb) [file 12916_2017_988_MOESM2_ESM.docx]

**Additional file 2. Deprescribing guideline**The following examples of dose-lowering schemes (table 1) were available for the general practitioners (GPs) to provide guidance when withdrawal of medication was attempted.

| **Table S1** Examples of dose-lowering schemes | |
| --- | --- |
| **Medication** | **Example of dose-lowering scheme (in mg, per week)** |
|  |  |
| Lipid-lowering drugs (in general) | Stop at once |
| Hydrochlorothiazide | 25 – 12.5 – 0 |
| Chlorthalidone | 25 – 12.5 – 0 |
| Nifedipine | 120 – 60 – 30 – 0 |
| Amlodipine | 10 – 5 – 0 |
| Metoprolol | 100 – 50 – 25 – 0 |
| Enalapril | 40 – 20 – 10 – 5 – 0 |
| Lisinopril | 30 – 20 – 10 – 5 – 0 |
| Losartan | 100 – 50 – 5 |
|  |  |

According to the research protocol, the GP or practice nurse monitored the patient when withdrawal of medication was attempted. In case antihypertensive drugs were deprescribed, blood pressure monitoring occured after four and 12 weeks and after six months; in case lipid-lowering drugs were deprescribed, lipid level monitoring was conducted after 12 weeks. After medication was stopped and monitoring had taken place according to the research protocol, patients were monitored according to the Dutch guideline for Cardiovascular Risk Management.[^1^](#_ENREF_1)

When monitoring patients according to the research protocol, in addition to blood pressure and lipid level measurement, the GP or practice nurse asked whether the patient experienced adverse effects. The GP or practice nurse always asked whether shortness of breath, oedema, or weight gain had occurred. The pulse was assessed to determine pace and regularity. According to the research protocol, the practice nurse had to consult the GP in case the patient: 1) did not feel well; 2) experienced symptoms of heart failure (shortness of breath, oedema); 3) gained >2 kg in body weight; 4) had a systolic blood pressure >180 mm Hg; 5) had a pulse rate >100/minute; 6) had an irregular pulse; 7) (possibly) experienced an adverse effect of withdrawal. The data safety monitoring board added a total cholesterol level >308.9 mg/dl (8 mmol/l) and a LDL-cholesterol level >193.1 mg/dl (5 mmol/l) to this list.

REFERENCES

1. Wiersma T, Smulders YM, Stehouwer CD, Konings KT, Lanphen J. [Summary of the multidisciplinary guideline on cardiovascular risk management (revision 2011)]. *Ned Tijdschr Geneeskd* 2012;156:A5104.
