# Supplementary material for: Deprescribing preventive cardiovascular medication in patients with predicted low cardiovascular disease risk in general practice – the ECSTATIC study: a cluster randomised non-inferiority trial
Source: BMC Med. 2018 Jan 11;16:5. doi: 10.1186/s12916-017-0988-0 (PMC5763574; doi:10.1186/s12916-017-0988-0)
Supplement: Supplementary file 3 — Cost-effectiveness analysis. Table S2. Costs (in £) for the preparation of the intervention, per selected patient. Table S3. Costs (in £) and QALYs per patient in usual care group and intervention group. Figure S1. EuroQol Utility at t = 0, t = 3, t = 6, t = 12 and t = 24 in the usual care group and the intention. Figure S2. Cost-effectiveness acceptability curve showing the probability that an attempt to deprescribe preventive cardiovascular medication is cost-effective compared to usual care. (DOCX 66 kb) [file 12916_2017_988_MOESM3_ESM.docx]

**Additional file 3.** **Cost-effectiveness analysis**

| **Table S2** Costs (in £) for the preparation of the intervention, per selected patient^a^ | | | |
| --- | --- | --- | --- |
| **Activity** | **Time investment (in minutes)** | **Costs per 10 minutes^b^** | **Costs** |
|  |  |  |  |
| Workshop GP | 150 | 28 | 420 |
| Workshop practice nurse | 150 | 9 | 140 |
| Patient group selection by GP | 120 | 28 | 336 |
|  |  |  |  |
| Group costs per GP practice | 420 |  | 896 |
| Group costs per selected patient (n=160)^a^ | 3 |  | 6 |
| Individual preparation of invitation/consultation | 10 | 28 | 28 |
|  |  |  |  |
| **Costs per selected patient** |  |  | **34** |
|  |  |  |  |
| Abbreviations: GP denotes general practitioner. ^a^ Based on the number of patients invited to participate in the ECSTATIC trial, with an estimated GP practice size of 2168 (NZa 2016, URL: https://www.lhv.nl/uw-beroep/over-de-huisarts/kerncijfers-huisartsenzorg).  ^b^ Obtained from Dutch guidelines for economic evaluations, at the price level of 2015.^21^ | | | |

| **Table S3** Costs (in £) and QALYs per patient in usual care group and intervention group | | | | | | | | | | | |  |
| --- | --- | --- | --- | --- | --- | --- | --- | --- | --- | --- | --- | --- |
|  | | |  | | | | | | | **Usual care group**  (n=575) | **ITT intervention group**  (n=492^a^) | P value |
|  | | |  | | | | | | |  |  |  |
| Preparation for intervention | | |  | | | | | | |  |  |  |
|  | Year 1 | | | | | | | | | 0 | 34 | - |
|  | Year 2 | | | | | | | | | 0 | 0 | - |
| General practice consultations | | | | | | | |  | |  |  |  |
|  | Year 1 | | | | | | | | | 170 | 222 | <0.01 |
|  | Year 2 | | | | | | | | | 179 | 171 | 0.58 |
| Preventive cardiovascular medication | | | | | | | |  | |  |  |  |
|  | Year 1 | | | | | | | | | 36 | 20 | <0.01 |
|  | Year 2 | | | | | | | | | 33 | 21 | <0.01 |
| Laboratory^b^ | | | | |  | | | | |  |  |  |
|  | Year 1 | | | | | | | | | 32 | 33 | 0.67 |
|  | Year 2 | | | | | | | | | 31 | 29 | 0.34 |
| Other healthcare^c^ | |  | | | | | | | |  |  |  |
|  | Year 1 | | | | | | | | | 508 | 472 | 0.55 |
|  | Year 2 | | | | | | | | | 617 | 606 | 0.93 |
|  | | | |  | | | | | |  |  |  |
| Total primary care specific costs^d^ | | | | | | | | | |  |  |  |
|  | Year 1 | | | | | | | | | 239 | 309 | <0.01 |
|  | Year 2 | | | | | | | | | 243 | 220 | 0.19 |
| **Total primary care specific costs over two years^e^** | | | | | | | | |  | 482 | 528 | 0.19 |
|  | | | |  | | | | | |  |  |  |
| Total healthcare costs^f^ | | | |  | | | | | |  |  |  |
|  | Year 1 | | | | | | | | | 742 | 787 | 0.49 |
|  | Year 2 | | | | | | | | | 861 | 824 | 0.77 |
| **Total healthcare costs over two years^e^** | | | | | |  | | | | 1607 | 1607 | 1.00 |
|  | | | | | |  | | | |  |  |  |
| QALYs | | | | | |  | | | |  |  |  |
|  | Year 1 | | | | | | | | | 0.870 | 0.879 | 0.38 |
|  | Year 2 | | | | | | | | | 0.874 | 0.879 | 0.58 |
| **Total QALYs^e^** | | | | | | |  | | | 1.743 | 1.759 | 0.45 |
|  |  | | | | | | | | |  |  |  |
| Abbreviations: QALYs denotes quality-adjusted life years; ITT denotes intention-to-treat. ^a^ One patient who died of unknown cause during follow-up without having attempted to have her preventive cardiovascular medication  deprescribed was left out in the analyses at 24 months. ^b^ Only cardiovascular management related ^c^ Costs of specialist and physical therapist consultations, use of home care, and hospitalisations. ^d^ Sum of costs of implementation of the intervention, general practice consultations, preventive cardiovascular medication, and  laboratory. ^e^ Sum of costs and QALYs in year 1 and year 2 do not exactly add up to the total costs over two years due to adjustment for cluster  randomization in the analyses.  ^f^ Primary care specific costs plus other costs. | | | | | | | | | | | | |

**Figure S1** EuroQol Utility at t=0, t=3, t=6, t=12, and t=24 in the usual care group and the intention
to treat population of the intervention group.
Abbreviations: ITT denotes intention-to-treat.
Measurements at t=0 were performed at the first visit.


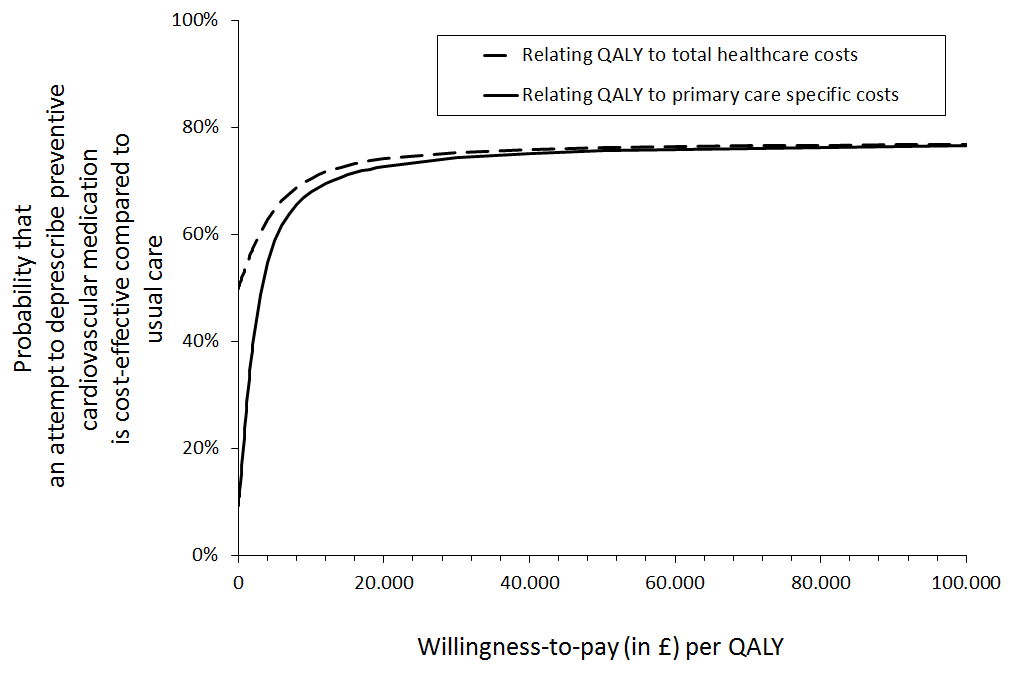


**Figure S2** Cost-effectiveness acceptability curve showing the probability that an attempt to

deprescribe preventive cardiovascular medication is cost-effective compared to usual care.
